# Expression Analysis and Mutational Status of Histone Methyltransferase *KMT2D* at Different Upper Tract Urothelial Carcinoma Locations

**DOI:** 10.3390/jpm11111147

**Published:** 2021-11-04

**Authors:** Ekaterina Laukhtina, Ursula Lemberger, Andreas Bruchbacher, Dafina Ilijazi, Stephan Korn, Florian Berndl, David D’Andrea, Martin Susani, Dmitry Enikeev, Eva Compérat, Shahrokh F. Shariat, Melanie R. Hassler

**Affiliations:** 1 Department of Urology, Comprehensive Cancer Center, Medical University of Vienna, 1090 Vienna, Austria; katyalaukhtina@gmail.com (E.L.); ursula.lemberger@meduniwien.ac.at (U.L.); andreas.bruchbacher@meduniwien.ac.at (A.B.); dafinailijazii@gmail.com (D.I.); stephan.korn@meduniwien.ac.at (S.K.); florian.berndl@meduniwien.ac.at (F.B.); david.dandrea@meduniwien.ac.at (D.D.); shahrokh.shariat@meduniwien.ac.at (S.F.S.); 2 Institute for Urology and Reproductive Health, Sechenov University, 119435 Moscow, Russia; dvenikeev@gmail.com; 3 Department of Pathology, Medical University of Vienna, 1090 Vienna, Austria; martin.susani@meduniwien.ac.at (M.S.); evacomperat@gmail.com (E.C.); 4 Karl Landsteiner Institute of Urology and Andrology, 1010 Vienna, Austria; 5 Department of Urology, Weill Cornell Medical College, New York, NY 10065, USA; 6 Department of Urology, University of Texas Southwestern, Dallas, TX 75390, USA; 7 Department of Urology, Second Faculty of Medicine, Charles University, 150 06 Prague, Czech Republic

**Keywords:** UTUC, urothelial cancer, KMT2 family, KMT2D, histone methylation

## Abstract

The gene coding for histone methyltransferase KMT2D is found among the top mutated genes in upper tract urothelial carcinoma (UTUC); however, there is a lack of data regarding its association with clinicopathologic features as well as survival outcomes. Therefore, we aimed to investigate *KMT2D* expression, mutation patterns, and their utility as prognostic biomarkers in patients with UTUC. A single-center study was conducted on tumor specimens from 51 patients treated with radical nephroureterectomy (RNU). Analysis of KMT2D protein expression was performed using immunohistochemistry (IHC). Customized next-generation sequencing (NGS) was used to assess alterations in *KMT2D* exons. Cox regression was used to assess the relationship of *KMT2D* protein expression and mutational status with survival outcomes. KMT2D expression was increased in patients with a previous history of bladder cancer (25% vs. 0%, *p* = 0.02). The NGS analysis of KMT2D exons in 27 UTUC tumors revealed a significant association between pathogenic *KMT2D* variants and tumor location (*p* = 0.02). Pathogenic *KMT2D* variants were predominantly found in patients with non-pelvic or multifocal tumors (60% vs. 14%), while the majority of patients with a pelvic tumor location (81% vs. 20%) did not harbor pathogenic *KMT2D* alterations. Both IHC and NGS analyses of *KMT2D* failed to detect a statistically significant association between KMT2D protein or *KMT2D* gene alteration status and clinical variables such as stage/grade of the disease or survival outcomes (all *p* > 0.05). *KMT2D* alterations and protein expression were associated with UTUC features such as multifocality, ureteral location, and previous bladder cancer. While KMT2D protein expression and *KMT2D* mutational status do not seem to have prognostic value in UTUC, they appear to add information to improve clinical decision-making regarding the type of therapy.

## 1. Introduction

Upper tract urothelial cell carcinoma (UTUC) is a rare disease with often a poor prognosis [1]. Indeed, two thirds of all cases are already detected at advanced tumor stages [2,3]. For risk stratification, clinicians use established clinicopathological factors such as multifocality, tumor size, tumor grade, cytology, invasiveness on CT urography, previous bladder cancer, variant histology, and concomitant hydronephrosis [4,5,6,7]. This standardized risk stratification helps in the decision-making process between kidney-sparing therapy and radical nephroureterectomy (RNU) [4]. However, current risk models and diagnostic approaches do not capture the biologic and clinical behavior of UTUC accurately [8]. Insights into the molecular mechanisms underlying UTUC could provide a rationale for different treatment approaches as well as uncovering novel biomarkers for tailored therapy development [9].

It is believed that epigenetic changes affect carcinogenesis, and mutations in epigenetic modifiers frequently show cancer-specific alteration patterns [10]. One of the players among epigenetic modifiers, with an assumed role in urothelial cancer development, is the histone-lysine N-methyltransferase 2 (*KMT2*) family of histone methylases comprising KMT2A, KMT2B, KMT2C, and KMT2D [11,12]. Recent studies reported that alterations in the gene coding for histone methyltransferase *KMT2D* are early events of urothelial carcinogenesis [13] along with several other malignancies, including breast cancer, and pancreatic ductal and lung adenocarcinoma [14,15,16,17]. *KMT2D* is, moreover, among the top mutated genes in both upper and lower tract urothelial carcinoma [18,19]. Despite *KMT2D’s* putative significant role in urothelial cancer development, there is a lack of data regarding its function and association with clinicopathologic features and survival in UTUC patients.

Therefore, we aimed to investigate KMT2D protein expression, mutation patterns, and prognostic value in UTUC patients using immunohistochemistry (IHC) and targeted next-generation sequencing (NGS).

## 2. Materials and Methods

### 2.1. Data Source and Patient Cohort

This retrospective single-center study included a consecutive cohort of 51 patients treated with RNU for UTUC at the Department of Urology of the Medical University of Vienna between 1993 and 2014. Lymphadenectomies were performed at the surgeons’ discretion. Adjuvant chemotherapy was administered to patients at the clinicians’ discretion based on tumor stage and overall health status. No patient received adjuvant radiotherapy.

### 2.2. Pathologic Review and Follow-Up

All surgical specimens were processed according to standard pathological procedures. Genitourinary pathologists assigned a tumor grade according to the 2004 WHO grading system. The pathological stage was reassigned according to the 2002 American Joint Committee on Cancer TNM staging system. Specimens from FFPE material for IHC and DNA isolation were obtained after approval from the institutional review board. Only FFPE tumor samples with the presence of more than 80% of tumor tissue per sample were used for staining and DNA isolation.

Clinical and radiological follow-ups were performed in accordance with institutional protocols and current guidelines. Routine follow-up usually included physical examination, radiological imaging, and urinary cytology at three and six months, and then yearly. Disease-specific survival (DFS) time was calculated from the date of RNU to disease recurrence/progression or last follow-up. Cause of death was abstracted from medical charts and/or from death certificates [20]. Overall survival (OS) time was calculated from the date of RNU to death or last follow-up.

### 2.3. Immunohistochemistry

An analysis of KMT2D expression at the protein level was performed by IHC. Formalin-fixed, paraffin-embedded slides were processed according to the standard methods for IHC. Heat-induced antigen retrieval was performed with citrate buffer (pH = 6). Staining was accomplished with a KMT2D antibody (1:500 dilution, abcam ab224156, Lot GR3254038-2) using the Histostain Plus Broad Spectrum Novex Protein Kit, Life Technology (Carlsbad, CA, USA) (Lot 1954379A), according to the manufacturer’s instructions. AEC Single Solution Life Technology (Lot 1936895A) was used for development, and Hematoxylin Solution, Merck (Whitehouse Station, NJ, USA) (Lot HX86017674), was used for counterstaining. The histoscore (H-score) and staining intensity were evaluated by an experienced uropathologist. Microscopy was performed on a Zeiss Axio in 20× magnification for normal and tumor areas, and a 10× objective was used for normal/tumor takes.

### 2.4. Next-Generation Sequencing

Genomic DNA was purified from formalin-fixed, paraffin-embedded tissue using the Gene Read DNA FFPE Kit, Quiagen (Shanghai, China) (CatNr. 180134, Lot 160040260). The DNA quality was quantified with the Agilent High Sensitivity DNA Kit, Agilent (Palo Alto, CA, USA) (CatNr. 5067-4626, Lot YB26BK50, and CatNr. 5067-1508, Lot 1536) on the Bioanalyzer 2100 by Agilent. Library preparation was performed according to the manufacturer’s instructions, processing 8.5 ng DNA per sample with the Ion AmpliSeq Customized KMT2 DNA Panel (IAD177281_167), Ion AmpliSeq Library Kit Plus (Lot 1970973), and Ion Xpress Barcode Adaptors (Lot 157170) from Ion Torrent, Life Technologies. Chip loading was performed on an Ion Chef System using Ion S5 Chef Supplies (Lot 2035927), Ion S5 Chef Solutions (Lot 2049981), and two Ion 540 Chips (Lot Q0UU58E). The library was quantified using the Ion Library TaqMan Quantification Kit (Lot 00717037) on a Quantstudio 7Flex, and sequencing was accomplished using an Ion S5 XL device, all from Life Technologies.

Data were processed on the Ion Torrent Server via Ion Reporter (Thermofisher, Waltham, MA, USA), Torrent Variant Caller 5.2. Variants were filtered and analyzed according to clinical standards in cooperation with the Institute of Pathology, Medical University of Vienna. Only mutations with an allele ratio of 2% to 100%, a minor allele frequency of at least 5%, and a read coverage of >200 were included. Furthermore, only missense, nonsense, stoploss, and frameshift mutations were further analyzed.

Within the Ion Reporter software annotation tools, SIFT, PolyPhen-2, and ClinVar were implemented and directly analyzed. Mutations of interest were directly compared with the ClinVar database (https://www.ncbi.nlm.nih.gov/clinvar (accessed on 17 August 2021)). The PolyPhen-2 score was directly analyzed by the PolyPhen-2 prediction tool from Harvard University (website: http://genetics.bwh.harvard.edu/pph2/ (accessed on 17 August 2021)) [21]. Mutations with a PolyPhen-2 score of 0.0 to 0.15 were predicted as benign, a score from 0.15 to 0.85 as possibly damaging, and a score of >0.85 as confidently damaging. The SIFT score within the Ion Reporter software was directly analyzed using the PROVEAN Genome Variants annotation tool provided by JCVI J. Craig Venter Institute (website: http://provean.jcvi.org/index.php (accessed on 17 August 2021)) [22]. Mutations with a SIFT score between 0.0 and 0.05 were considered deleterious. Mutations with a SIFT score between 0.05 and 1.0 were predicted to be tolerated (benign).

A scoring system combining SIFT and PolyPhen-2 scores with the ClinVar database was established, with the ClinVar database as a main reference. In cases of mutations without any ClinVar database information, the SIFT and PolyPhen-2 scores were combined to determine potential pathogenic mutations. At least one of the above-mentioned scores contained a result for pathogenicity to register a potential pathogenic mutation. In cases of divergent results, the mutation was classified as uncertain.

### 2.5. Statistical Analysis

The report of categorical variables included frequencies and proportions. Continuous variables were reported as medians or means and interquartile ranges (IQRs) or ranges. The correlation and intensity of KMT2D gene alteration and protein expression patterns with clinical data were statistically analyzed using Wilcoxon rank-sum and Fisher’s exact tests, as appropriate. Cox regression analyses were used to assess the correlation of KMT2D protein expression and KMT2D mutational status with survival outcomes such as DFS and OS. The risk of survival was expressed as the hazard ratio (HR) and 95% confidence interval (95% CI). Kaplan–Meier survival curves were used to depict the association between KMT2D expression and KMT2D gene alteration and survival. The log-rank test was used to determine the statistical difference between groups. All reported *p*-values were two-sided, and statistical significance was set at 0.05. All statistical analyses were performed using R Version 4.0.4.

## 3. Results

A total of 51 UTUC patients treated with RNU were included. The median age of the entire cohort was 73 years (IQR 60–79). The patient characteristics are shown in Table 1. A total of 13 out of the 51 cases received chemotherapy as an adjuvant or in a palliative setting. The median survival and duration of follow-up for consecutively recruited patients were 29 months (IQR 7.5–79.5). The median follow-up of patients alive was 22.5 months (IQR 8.0–70.5).

### 3.1. KMT2D Expression

**Figure 1:**
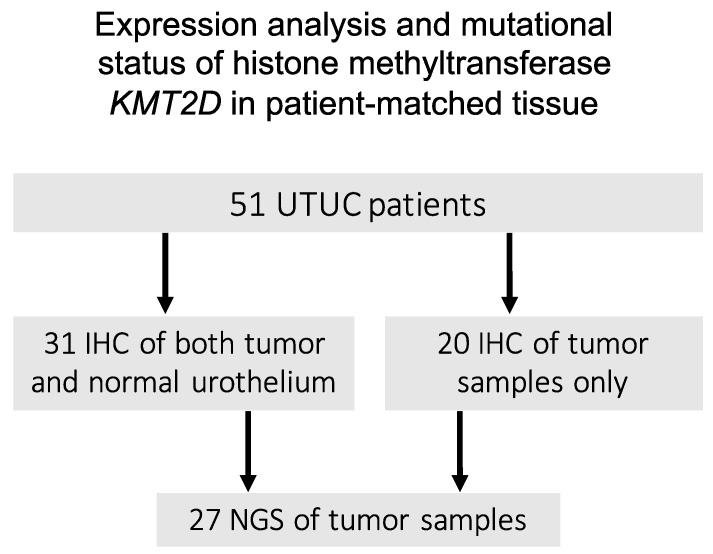
Overview of tissue sample processing from 51 upper tract urothelial carcinoma (UTUC) patients.

The H-score from tumor tissue and normal urothelium could be assessed in 31 patients, while in 20 patients, only tumor tissue was available for evaluation (Figure 1). According to the IHC analysis, the median H-score in tumor tissue was 90 (range: 0–300). Tumors with an H-score of >0 were deemed KMT2D-positive. A total of 19 tumor specimens (37.3%) showed negative KMT2D expression, whereas 32 tumor specimens showed positive KMT2D expression (62.7%).

**Figure 2:**
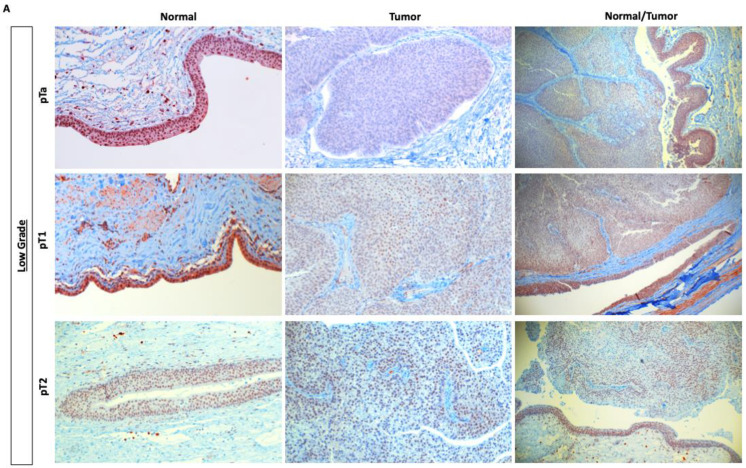

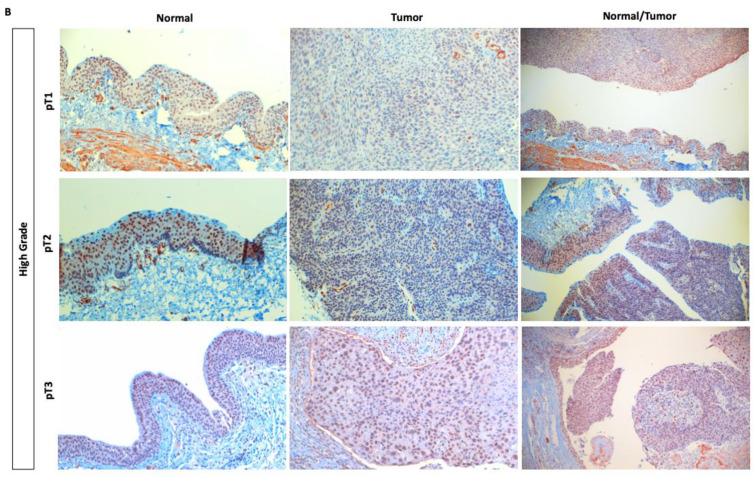
Representative images for differential protein expression of KMT2D in (**A**) low-grade upper tract urothelial carcinoma samples (pTa, pT1, and pT2) and (**B**) high-grade upper tract urothelial carcinoma samples (pT1, pT2, and pT3) in normal and tumor tissue. Nuclear expression of KMT2D increases with stage in tumor tissue.

When looking at protein expression only in patient samples with both normal urothelium and tumor tissue available, we found a higher mean H-score in normal tissue compared to tumor tissue (127.3 vs. 74.5) in low-grade (LG) samples (Figure 2A). In high-grade (HG) samples (Figure 2B), there was no difference in nuclear expression between normal urothelium and malignant tissue (mean values: 46.5 vs. 47.5). For LG tumor samples, the nuclear expression of KMT2D was low in pTa but increased with stage. We did not observe similar patterns in HG tumors.

Significantly increased KMT2D expression was detected in patients with a previous history of bladder cancer (25% vs. 0%, *p* = 0.02). No significant association was observed between KMT2D expression and age, sex, pathological T stage, histological grade, tumor location and side, lymph node involvement, metastases, or site of recurrence (all *p* > 0.05).

**Figure 3:**
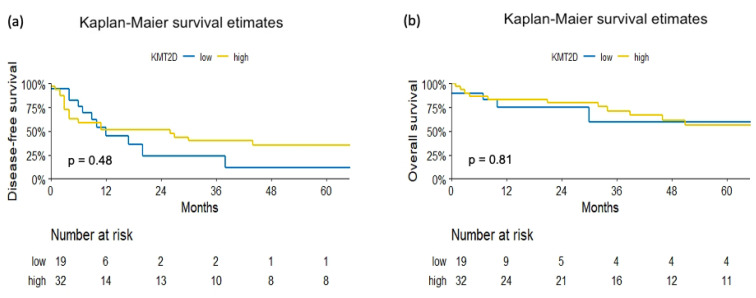
Kaplan–Meier analysis for (**a**) disease-free survival and (**b**) overall survival in 51 patients treated with radical nephroureterectomy for upper tract urothelial carcinoma. Stratification was carried out according to KMT2D protein expression.

When analyzing survival outcomes, KMT2D expression was not associated with DFS (HR 0.77, 95% CI: 0.36–1.67, *p* = 0.51) or OS (HR 0.89, 95% CI: 0.36–2.19, *p* = 0.8) (Figure 3). In patients with positive compared to negative KMT2D expression, the 3-year DFS was 40% (95% CI 25–63%) vs. 24% (95% CI 8–71%), respectively. The 3-year OS for patients with positive KMT2D expression was 71% (95% CI 56–91%) compared to 60% (95% CI 35–100%) for those with negative KMT2D expression.

### 3.2. KMT2D Alterations

To identify somatic mutations, NGS was successfully performed on 51 tumor samples from patients who had tumor-only and both tumor and normal tissues. A total of 22 samples were excluded due to the small number of reads, and 2 samples were excluded due to the presence of a high number of mutations (up to 100 mutations) per sample. From the remaining 27 patients, 5 specimens (18.5%) showed pathogenic *KMT2D* variants, whereas 22 specimens showed non-pathogenic *KMT2D* variants (81.5%). Overall, seven pathogenic/likely pathogenic variants were identified in our study. Detailed information on the validated variants with the ID of the pathogenic variants is presented in Figure S1. NGS analysis of UTUC tumors of different stages and grades revealed a significant association between the *KMT2D* variant and tumor location (*p* = 0.02). Pathogenic *KMT2D* variants were predominantly found in patients with non-pelvic or multifocal tumors (60% vs. 14%), while the majority of patients with a pelvic tumor location (81% vs. 20%) did not harbor *KMT2D* alterations. No significant association was noted in our NGS cohort with respect to the following clinicopathological features: age, sex, pathological T stage, histological grade, tumor side, lymph node involvement, metastases, site of recurrence, and previous history of bladder cancer (all *p* > 0.05).

**Figure 4:**
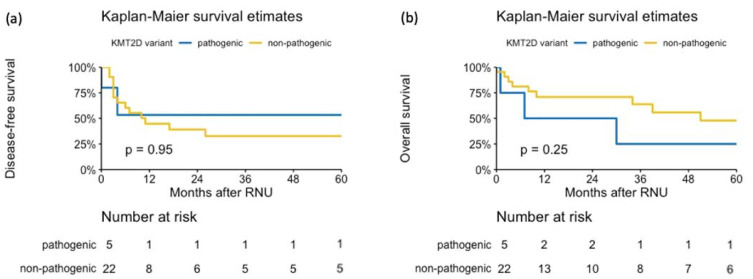
Kaplan–Meier analysis for (**a**) disease-free survival and (**b**) overall survival in 27 patients treated with radical nephroureterectomy for upper tract urothelial carcinoma. Stratification was carried out according to *KMT2D* gene alteration.

When analyzing survival outcomes, *KMT2D* alterations were neither associated with DFS (HR 1.06, 95% CI: 0.24–4.72, *p* = 0.94) nor OS (HR 0.47, 95% CI: 0.13–1.74, *p* = 0.26) (Figure 4). In patients with pathogenic compared to non-pathogenic *KMT2D* variants, the 3-year DFS was 53% (95% CI 21–100%) vs. 33% (95% CI 17–63%), respectively. The 3-year OS for the patients with pathogenic *KMT2D* variants was 25% (95% CI 46–100%) compared to 64% (95% CI 45–90%) for those with non-pathogenic *KMT2D* variants.

## 4. Discussion

According to our knowledge, this study is the first to investigate KMT2D expression along with alterations in UTUC. Our results show that both KMT2D protein expression and *KMT2D* alterations were not significantly associated with clinical variables such as stage or grade of the disease. *KMT2D* alterations and expression also failed to emerge as a prognostic marker for UTUC. However, pathogenic *KMT2D* alterations and expression were associated with features of clinically aggressive UTUC including multifocality, ureteral location, and previous bladder cancer.

The KMT2 family member KMT2D, which regulates the H3K4me methylation landscapes predominantly at enhancers, has been implicated in the development of cancer by dysregulation of enhancer activity and subsequent disruption of normal development programs [23,24,25,26]. In urothelial carcinoma, *KMT2D* has been found among the top mutated genes in several genomic characterization studies; it seems to be an early event in the pathogenesis of UTUC rather than a driver of disease progression [18,19,27,28]. Indeed, in our study, *KMT2D* alterations were not significantly associated with clinical variables such as stage or grade of the disease.

However, pathogenic *KMT2D* variants were predominantly found in patients with non-pelvic and multifocal tumors, while the majority of patients with a pelvic tumor location did not harbor *KMT2D* alterations. This finding is in agreement with previous studies reporting that *KMT2C* and *KMT2D* were more frequently altered in ureteral than in renal pelvic tumors [26]. Several studies have shown that the initial tumor location is a prognostic factor, as patients with ureteral and/or multifocal tumors seem to have a worse prognosis than patients diagnosed with renal pelvic tumors [29,30]. Additionally, according to our results, significantly increased KMT2D protein expression was detected in patients with a previous history of bladder cancer, which has been reported as a risk factor for bladder recurrence after RNU [31]. Furthermore, in LG disease, nuclear KMT2D protein expression was lower in tumors compared to adjacent normal urothelium, whereas in HG disease, reduced protein expression was observed both in tumor and adjacent normal urothelium, with no significant difference between them. We hypothesize that this reduced expression also found in normal urothelium may be a sign of a possible field effect in HG disease [13]. Thus, *KMT2D* alterations and expression were associated with features of biologically aggressive UTUC including multifocality, ureteral location, a cancer field effect, and previous bladder cancer. This association of reduced KMT2D protein expression and pathogenic alterations with aggressive clinicopathologic features could help physicians choose tailored perioperative treatment strategies with intensified therapy in those most likely to benefit from it.

In addition to the prediction of biologically aggressive disease, prognostication of survival outcomes also allows for personalized decision-making. According to our analyses, neither KMT2D protein expression nor mutational status was associated with survival outcomes. These findings might be due to the lack of statistical power of our analysis because of our limited sample size. However, data from a previous study comprising survival outcomes of 71 patients (22 with a pathogenic alteration in *KMT2D*) also do not show significantly different outcomes when stratified according to alteration status [27]. For further conclusions, *KMT2D* alteration and survival outcomes in UTUC have to be studied in large-scale cohorts.

Interestingly, the only feature significantly different between publicly available TCGA samples with and without pathogenic *KMT2D* alterations was the mutation count, indicating a higher mutational burden in samples with pathogenic *KMT2D* alterations. As mutational burden has been reported to be associated with better response to immune checkpoint inhibitor (ICI) therapy, this could mean that tumors with *KMT2D* alterations may be more likely to respond to ICI treatment. Indeed, it has been reported that the alteration status of *KMT2* family members may serve as a potential predictor of favorable ICI response in multiple cancers [32,33]. Tumors with *KMT2D* mutations, as a major modulator of immune checkpoint blockade, were characterized by increased immune infiltration [34]. Future studies should assess this theory in the context of urothelial carcinoma patients receiving ICI therapies to evaluate whether *KMT2D* could serve as a predictive biomarker for ICI response.

The prevailing hypothesis is that alterations in KMT2 function contribute to carcinogenesis through the modification of histone methylation patterns; thus, novel therapeutic agents targeting other histone demethylases may be an option to inhibit disease recurrence and/or progression in tumors with *KMT2D* alterations [35,36]. Given that the data from UTUC patients from the TCGA show that *KMT2D* alterations are co-expressed with fibroblast growth factor receptor (*FGFR3*) alterations in more than half of the cases, a plausible treatment strategy may also be a combination of FGFR3 inhibitors with histone demethylase inhibitors [37]. However, this concept needs to be assessed in patients with treatment-naïve or previously treated advanced UTUC requiring systemic therapy.

The present study is, to our knowledge, the first to investigate KMT2D expression by IHC as well as the correlation of KMT2D protein expression and mutational status with survival outcomes. Nevertheless, our study is limited by its retrospective design and relatively small sample size, which limit the power of the study. However, several factors strengthen our study, as tissue samples were obtained from a single institution. Additionally, KMT2D expression and alterations in tumor tissue and normal urothelium were assessed at a single time point in samples obtained from RNU. Further well-designed studies should be conducted to test the prognostic and predictive biomarker potential of KMT2D expression and *KMT2D* alterations.

## 5. Conclusions

KMT2D expression and mutational status did not emerge as a prognostic marker for UTUC; however, KMT2D alterations and expression were associated with features of clinically aggressive UTUC such as multifocality, ureteral location, and previous bladder cancer. Therefore, determination of KMT2D expression and KMT2D alteration may hold potential for identifying the best treatment strategy for UTUC patients.

## Figures and Tables

**Table 1:** Clinicopathologic characteristics of patients treated with radical nephroureterectomy for upper tract urothelial carcinoma in overall patient population as well as in the groups stratified by KMT2D expression and *KMT2D* gene alteration.

	IHC				NGS			
	Overall	Stratified by KMT2D Staining			Overall	Stratified by KMT2D Variant		
	N = 51	Negative, N = 19	Positive, N = 32	*p*-Value	N = 27	Pathogenic, N = 5	Non-Pathogenic, N = 22	*p*-Value
Age	73 (60, 79)	73 (54, 78)	73 (63, 78)	0.7	71 (61, 80)	61 (45, 71)	73 (64, 80)	0.2
Sex				0.5				0.6
female	24 (47%)	10 (53%)	14 (44%)		12 (44%)	3 (60%)	9 (41%)	
male	27 (53%)	9 (47%)	18 (56%)		15 (56%)	2 (40%)	13 (59%)	
Pathological tumor stage				0.8				>0.9
pT1	14 (27%)	6 (32%)	8 (25%)		8 (30%)	2 (40%)	6 (27%)	
pT2	6 (12%)	1 (5.3%)	5 (16%)		1 (3.7%)	0 (0%)	1 (4.5%)	
pT3	20 (39%)	8 (42%)	12 (38%)		11 (41%)	2 (40%)	9 (41%)	
pT4	7 (14%)	2 (11%)	5 (16%)		5 (19%)	1 (20%)	4 (18%)	
pTa	4 (7.8%)	2 (11%)	2 (6.2%)		2 (7.4%)	0 (0%)	2 (9.1%)	
N stage				0.3				0.5
pN0	8 (16%)	2 (11%)	6 (19%)		4 (15%)	0 (0%)	4 (18%)	
pN1	6 (12%)	2 (11%)	4 (12%)		5 (19%)	2 (40%)	3 (14%)	
pN2	4 (7.8%)	0 (0%)	4 (12%)		3 (11%)	0 (0%)	3 (14%)	
pNx	33 (65%)	15 (79%)	18 (56%)		15 (56%)	3 (60%)	12 (55%)	
M stage				0.8				>0.9
M0	45 (88%)	16 (84%)	29 (91%)		23 (85%)	5 (100%)	18 (82%)	
M1	3 (5.9%)	2 (11%)	1 (3.1%)		2 (7.4%)	0 (0%)	2 (9.1%)	
Mx	3 (5.9%)	1 (5.3%)	2 (6.2%)		2 (7.4%)	0 (0%)	2 (9.1%)	
Grade				0.7				>0.9
HG	39 (76%)	14 (74%)	25 (78%)		19 (70%)	4 (80%)	15 (68%)	
LG	12 (24%)	5 (26%)	7 (22%)		8 (30%)	1 (20%)	7 (32%)	
Location				0.8				**0.02**
multifocal	9 (18%)	3 (16%)	6 (19%)		6 (22%)	3 (60%)	3 (14%)	
pelvis	34 (67%)	13 (68%)	21 (66%)		19 (70%)	1 (20%)	18 (82%)	
proximal	6 (12%)	3 (16%)	3 (9.4%)		2 (7.4%)	1 (20%)	1 (4.5%)	
distal	2 (3.9%)	0 (0%)	2 (6.2%)					
Tumor side				0.5				0.6
left	24 (47%)	10 (53%)	14 (44%)		14 (52%)	2 (40%)	12 (55%)	
right	27 (53%)	9 (47%)	18 (56%)		13 (48%)	3 (60%)	10 (45%)	
Number of positive lymph nodes				>0.9				0.3
0	8 (53%)	2 (67%)	6 (50%)		4 (44%)	0 (0%)	4 (57%)	
1	4 (27%)	1 (33%)	3 (25%)		3 (33%)	2 (100%)	1 (14%)	
3	2 (13%)	0 (0%)	2 (17%)		1 (11%)	0 (0%)	1 (14%)	
5	1 (6.7%)	0 (0%)	1 (8.3%)		1 (11%)	0 (0%)	1 (14%)	
Chemotherapy	13 (26%)	3 (17%)	10 (31%)	0.3	9 (35%)	1 (25%)	8 (36%)	>0.9
Metastasis	19 (37%)	6 (32%)	13 (41%)	0.5	13 (48%)	2 (40%)	11 (50%)	>0.9
History of bladder cancer	8 (16%)	0 (0%)	8 (25%)	**0.02**	2 (7.4%)	0 (0%)	2 (9.1%)	>0.9
Recurrence	22 (43%)	7 (37%)	15 (47%)	0.5	11 (41%)	1 (20%)	10 (45%)	0.6
Site of recurrence				>0.9				0.6
bladder	19 (37%)	7 (37%)	12 (38%)		9 (33%)	1 (20%)	8 (36%)	
no	32 (63%)	12 (63%)	20 (62%)		18 (67%)	4 (80%)	14 (64%)	
Patient died	23 (45%)	7 (37%)	16 (50%)	0.4	14 (52%)	3 (60%)	11 (50%)	>0.9
Months to Death/Censor	29 (8, 80)	10 (4, 26)	36 (16, 102)	**0.036**	18 (6, 60)	7 (1, 30)	20 (8, 64)	0.2
Median (IQR); n (%)								

## Supplementary Materials

The following is available online at https://www.mdpi.com/article/10.3390/jpm11111147/s1: Figure S1: Validated variants.
